# Optimal duration and timing of basic-life-support-only intervention for patients with out-of-hospital cardiac arrest

**DOI:** 10.1038/s41598-024-56487-3

**Published:** 2024-03-13

**Authors:** Yutaka Takei, Gen Toyama, Tsukasa Takahashi, Kentaro Omatsu

**Affiliations:** Graduate School of Health and Welfare, Niigata University of Health and Welfare, 1398 Shimami-Cho, Kita-Ku, Niigata, 950-3198 Japan

**Keywords:** Basic life support, Epinephrine, Emergency medical service, Out-of-hospital cardiac arrest, On-scene, Return of spontaneous circulation, Outcomes research, Health care

## Abstract

To elucidate the relationship between the interval from cardiopulmonary resuscitation initiation to return of spontaneous circulation (ROSC) and neurologically favourable 1-month survival in order to determine the appropriate duration of basic life support (BLS) without advanced interventions. This population-based cohort study included patients aged ≥ 18 years with 9132 out-of-hospital cardiac arrest of presumed cardiac origin who were bystander-witnessed and had achieved ROSC between 2018 and 2020. Patients were classified into two groups based on the resuscitation methods as the “BLS-only” and the “BLS with administered epinephrine (BLS-AE)” groups. Receiver operating characteristic (ROC) curve analysis indicated that administering BLS for 9 min yielded the best neurologically outcome for patients with a shockable rhythm [sensitivity, 0.42; specificity, 0.27; area under the ROC curve (AUC), 0.60] in the BLS-only group. Contrastingly, for patients with a non-shockable rhythm, performing BLS for 6 min yielded the best neurologically outcome (sensitivity, 0.65; specificity, 0.43; AUC, 0.63). After propensity score matching, multivariate analysis revealed that BLS-only resuscitation [6.44 (5.34–7.77)] was associated with neurologically favourable 1-month survival. This retrospective study revealed that BLS-only intervention had a significant impact in the initial minutes following CPR initiation. Nevertheless, its effectiveness markedly declined thereafter. The optimal duration for effective BLS-only intervention varied depending on the patient's initial rhythm. Consequently, advanced interventions should be administered within the first few minutes to counteract the diminishing effectiveness of BLS-only intervention.

## Introduction

The survival rate from out-of-hospital cardiac arrests (OHCA) widely varies among countries. High-quality cardiopulmonary resuscitation (CPR) with minimal interruption of chest compressions increases the survival chances of patients with OHCA^1^. Moreover, maintaining the quality of chest compressions increases the likelihood of achieving a prehospital return of spontaneous circulation (ROSC)^2^, which is associated with favorable neurological outcomes. A previous study reported a negative correlation between the time of on-scene emergency medical service (EMS) arrival with ROSC achievement and survival^3^, with the yield of survivors per minute of resuscitation declining after 8 min.

For adult patients in cardiac arrest, epinephrine administration in the early stage and after the third shock is recommended for non-shockable and shockable rhythms, respectively^4^. Another study showed that administering epinephrine within 10 min after scene arrival was associated with improved survival among patients with OHCA^5^. However, the required intravenous access and other advanced interventions may negatively impact the quality of chest compressions^6^. Additionally, simulation studies have suggested that introducing advanced life support can decrease the chest compression fraction^7^. To address these challenges and maintain CPR quality, a teamwork approach that prioritizes CPR and defibrillation within the first 6–8 min after scene arrival has been shown to improve outcomes in several regions^8^.

Based on this evidence, it is recommended that EMS personnel focus on providing chest compressions and defibrillation within minutes of scene arrival before initiating advanced interventions to maintain the quality of resuscitation. However, the optimal timing to transition from the basic life support (BLS) phase to the advanced intervention phase, including epinephrine administration, remains unclear. This study aimed to elucidate the relationship between the interval from CPR initiation to ROSC and neurologically favorable 1-month survival to determine the appropriate duration of BLS without advanced interventions.

## Methods

### Study design

This retrospective, population-based cohort study included patients aged ≥ 18 years with OHCA of presumed cardiac origin who were bystander-witnessed and had achieved ROSC. The study followed all the methods that were performed in accordance with the relevant guidelines and regulations. This study protocol was approved by the Ethics Committee of the Niigata University of Health and Welfare (No. 19068-230602).

### Study population and settings

Japan has a population of approximately 127 million people, living in an area of 378,000 km^2^. As of 2018, Japan had 726 fire headquarters, 1719 fire stations, and 5215 ambulances. Each municipality primarily operates its fire headquarters, which falls under the jurisdiction of the Ministry of Internal Affairs and Communications. Although similar to first responder systems where specifically trained citizens rush to the scene of resuscitation, such systems are generally not present in Japan.

### Ambulance crew

In Japan, each ambulance crew comprises three members, with at least one specially trained emergency medical technician (EMT), who is referred to as a "paramedic" in other countries. The EMS comprises a one-tiered ambulance system that includes both BLS and advanced life support. EMTs are not permitted to terminate resuscitation in the field until the patient exhibits obvious post-mortem changes. EMTs are responsible for securing an intravenous route and airway access using a supraglottic airway device in patients with cardiac arrest. Furthermore, specially trained EMTs administer epinephrine and intubate patients in cardiac arrest, as well as administer glucose solution to patients before cardiac arrest or administer fluids to correct shock in patients based on the medical director's direct instructions. The EMTs commence ventilation using a bag-valve-mask device. If ventilation is expected to be difficult with the standard procedure, they liaise with the medical director. Following this, they perform tracheal intubation or use a supraglottic device based on the medical director's instructions. The use of mechanical chest compression devices is at the discretion of each EMT. However, similar to other studies, the specific timing of their use is not reported^9^ and this information is not included in the database.

### Data collection

This nationwide Japanese study included baseline data obtained for 18,889,552 EMS-transported cases and 379,845 OHCA cases handled by the Fire and Disaster Management Agency between January 2018 and December 2020 as the most recent three-year data. These data comprised all core data items recommended by the Utstein-style reporting guidelines^10^ for cardiac arrests, including sex, age, initial cardiac rhythm, bystander CPR status, resuscitation status by EMTs (BLS-only or BLS with epinephrine administration), date and time of cardiac arrest, level of hospital to which the patient was transported, time series related to EMS activities, ROSC, 1-month survival, and 1-month neurological status.

All patients were followed up for up to 1 month by the fire station staff. Further, 1-month neurological outcomes were assessed using the Cerebral Performance Category (CPC) scale, which ranges from 1 (good cerebral performance) to 5 (brain death)^11^. CPC categories 1 and 2 (disabled but independent) were defined as indicative of favorable neurological outcomes.

### Participants

**Figure 1:**
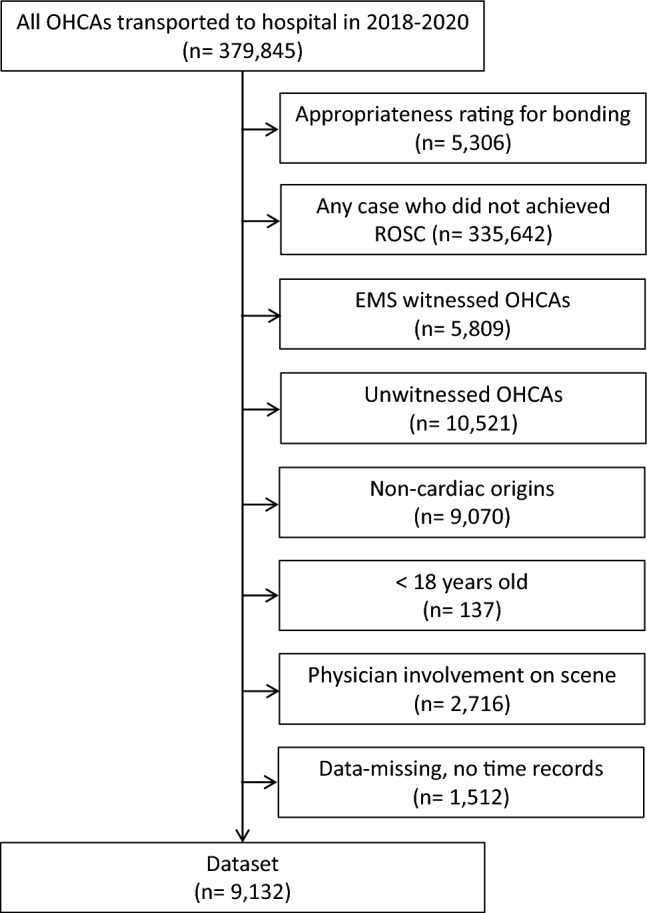
Flow. *OHCA* out-of-hospital cardiac arrest, *ROSC* return of spontaneous circulation, *EMS* emergency medical service.

We analyzed 9132 cases that met the inclusion criteria, involving bystander-witnessed cardiac events in patients aged ≥ 18 years of cardiac origin and without any physician involvement at the scene where ROSC was achieved. Cases that did not meet the inclusion criteria were excluded: cases with inconsistent binding between data sets (n = 5306), cases without successful ROSC (n = 335,642), cases witnessed by EMS staff (n = 5809), cases not witnessed by bystanders (n = 10,521), cases with a non-cardiac origin (n = 9070), cases involving patients aged < 18 years (n = 137), cases with physician involvement at the scene (n = 2716), and cases with missing data or no time records (n = 1512). (Fig. 1).

### Outcomes

The primary outcome was the neurologically favorable 1-month survival rate. The secondary outcome was the achievement of early ROSC.

### Statistical analysis

Patients were classified into two groups based on the resuscitation methods employed by EMTs as follows: the “BLS-only group” and the “BLS with administered epinephrine (BLS-AE) group”. Since defibrillation is recognized as a component of BLS in the resuscitation guidelines, the defibrillation intervention was included in the 'BLS-only group^12^.

The chi-square test was used to evaluate categorical variables, whereas the nonparametric Mann–Whitney U test was used for the two group comparisons to evaluate continuous variables. Nonparametric comparisons were due to the confirmation of a non-normal distribution through the use of a histogram for assessing the normality of the continuous variables. Receiver operating characteristic (ROC) curves were used to plot the ability of ROSC to predict outcomes as a function of increasing the pre-ROSC resuscitation time points. First, we performed a multiple logistic regression to identify factors related to the early CPR-to-ROSC interval, which was defined using a cut-off value. Finally, we performed propensity score matching to identify factors associated with the neurologically favorable 1-month survival. The propensity score prediction model comprised the following independent variables using multiple logistic regression analysis: resuscitation method (BLS-only or BLS-AE), age (continuous value), sex (male or female), initial cardiac rhythm (shockable or unshockable), bystander CPR status (provided or not provided), level of hospital to which the patient was transported, day of the week (weekday or weekend), and time of day [night-time (23:00–6:59) or daytime (7:00–22:59)] when the cardiac arrest occurred, the EMS response time (continuous value), time intervals between patients ‘ collapse and CPR initiation by EMS (continuous value), the on-scene time (continuous value) and the transport time (continuous value)^13^. The EMS response time was defined as the interval duration between the 119 calls and the on-scene arrival of the EMTs. The on-scene time was defined as the interval between the on-scene arrival of EMTs and the departure of the ambulance. Transport time was defined as the interval from ambulance departure to arrival at a hospital. All statistical analyses were performed using JMP Pro^®^ ver.17 for Windows (SAS Institute, Cary, NC, USA). Statistical significance was set at *p* < 0.05.

### Ethics approval and consent to participate

This study was approved by the Niigata University of Health and Welfare Ethics Committee (19068-230602).

### Inform consent

Due to the retrospective nature of the study, the need of informed consent was waived off by Niigata University of Health and Welfare Ethics Committee.

### Report format

All the methods were performed in accordance with relevant guidelines and regulations.

## Results

### Characteristics of patients with OHCA

The results are shown in Table 1. Compared with the BLS-AE group, the BLS-only group had a higher proportion of male patients (72.9% vs. 64.8%, *p* < 0.01), provision of bystander CPR (65.8% vs. 57.1%, *p* < 0.01), initial shockable rhythms (54.3% vs. 21.3%, *p* < 0.01), and transport to high-level hospitals (51.7% vs. 45.6%, *p* < 0.01).

**Table 1:** Characteristics of OHCAs between 2 groups.

Factors	BLS-only	BLS-AE	*p*-value
	(n = 4845)	(n = 4287)	
Day of the week			0.904
Weekend (Sat. to Sun.)	1476 (30.5%)	1311 (30.6%)	
Weekday (Mon. to Fri.)	3369 (69.5%)	2976 (69.4%)	
Time of the day			< 0.001
Nighttime (23:00 to 6:59)	839 (17.3%)	910 (21.2%)	
Daytime (7:00 to 22:59)	4006 (82.7%)	3377 (78.8%)	
Patient' age			
Median (25–75%)	70 years (59–80)	78 years (70–86)	< 0.001
Patient' sex			
Male	3530 (72.9%)	2776 (64.8%)	< 0.001
Female	1315 (27.1%)	1511 (35.2%)	
BCPR			< 0.001
Provided	3186 (65.8%)	2446 (57.1%)	
Not provided	1659 (34.2%)	1841 (42.9%)	
Initial ECG rhythms			< 0.001
Shockable	2631 (54.3%)	915 (21.3%)	
Non-shockable	2214 (45.7%)	3372 (78.7%)	
Level of hospital			< 0.001
Level 3	2507 (51.7%)	1953 (45.6%)	
Level 2 or 1	2338 (48.3%)	2334 (54.4%)	
Time factors, median (25–75%)			
EMS response time	8 min (7–10)	9 min (7–11)	< 0.001
Interval of Collapse-to-CPR	10 min (8–15)	10 min (7–13)	< 0.001
Interval of CPR-to-ROSC	6 min (4–11)	15 min (12–19)	< 0.001
Interval of CPR-to-Epinephrine	–	11 min (7–15)	–
Interval of Epinephrine -to-ROSC	–	6 min (3–10)	–
On-scene time	11 min (8–14)	14 min (11–17)	< 0.001
Transport time	10 min (7–14)	12 min (8–16)	< 0.001

*OHCA* out-of-hospital cardiac arrest, *BLS* basic life support, *BLS-AE* basic life support with administrated epinephrine, *BCPR* bystander cardiopulmonary resuscitation, *ECG* Electrocardiogram, *EMS* emergency medical service, *CPR* cardiopulmonary resuscitation, *ROSC* return of spontaneous circulation.

The EMS response time was defined as the time from EMS call to arrival at the patient.

Collapse-to-CPR interval was defined as the time from cardiac arrest being witnessed by a bystander to the initiation of CPR by EMS.

On-scene time was defined as the EMS arrival at the patient to the departure of an ambulance.

Transport time was defined as departure from an ambulance to arrival at a hospital.

Additionally, compared with the BLS-AE group, the BLS-only group had significantly longer time intervals between patients’ collapse and CPR initiation by EMS [median: 10 min (25–75%: 8–15) vs. 10 min (7–13), *p* < 0.01], shorter time intervals between EMT-administered CPR and ROSC achievement [6 min (4–11) vs. 15 min (12–19), *p* < 0.01] and a shorter on-scene time [11 min (8–14) vs. 14 min (11–17), *p* < 0.01].

Contrastingly, compared with the BLS-only group, the BLS-AE group had a higher proportion of OHCA occurrence at night (17.3% vs. 21.2%, *p* < 0.01) and older patients [70 years (59–80) vs. 78 years (70–86), *p* < 0.01]; furthermore, the BLS-AE group had a statistically significantly longer EMS response [8 min (7–10) vs. 9 min (7–11), *p* < 0.01] and transport times [10 min (7–14) vs. 12 min (8–16), *p* < 0.01].

### ROC curve and cut-off analysis results

**Figure 2:**
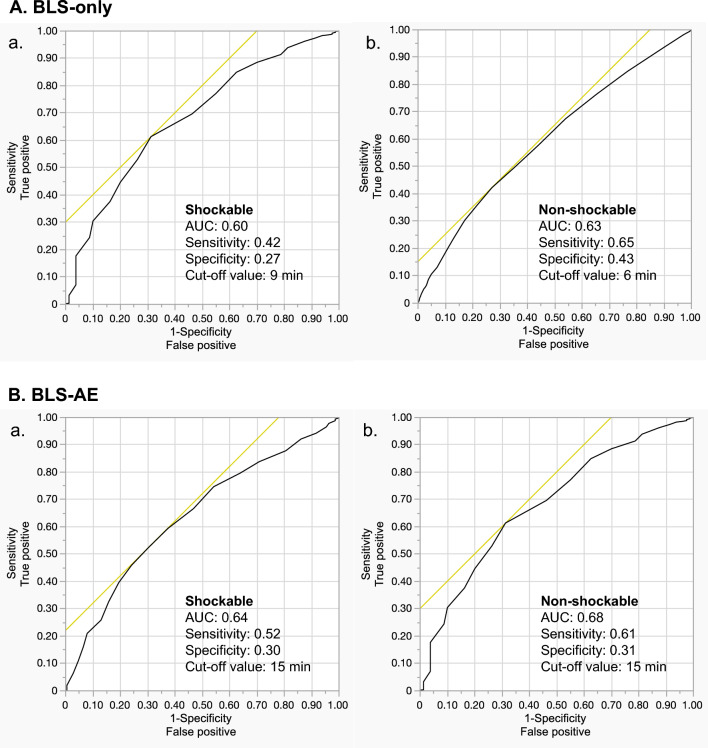
ROC curve and cut-off analysis. *BLS* basic life support, *AUC* area under the curve.

The neurologically favorable 1-M survival rates were 50.9% [2468/4845] for the BLS-only group and 6.9% [296/4287] for the BLS-AE group (*p* < 0.01). The results of the ROC curve and the cut-off analysis are shown in Fig. 2. The ROC curve analysis indicated that administering BLS for 9 min yielded the best neurologically favorable 1-M survival rate for patients with a shockable initial rhythm [sensitivity, 0.42; specificity, 0.27; area under the ROC curve (AUC), 0.60] in the BLS-only group. In contrast, for patients with a non-shockable initial rhythm, performing BLS for 6 min yielded the best neurologically favorable 1-M survival rate (sensitivity, 0.65; specificity, 0.43; AUC, 0.63).

Among patients who received epinephrine, administering BLS for 15 min yielded the best neurologically favorable 1-M survival rate for patients with shockable (sensitivity, 0.52; specificity, 0.30; AUC, 0.64) and non-shockable (sensitivity, 0.61; specificity, 0.31; AUC, 0.68) rhythms.

### Comparison of survival curves according to duration of CPR Until ROSC

**Figure 3:**
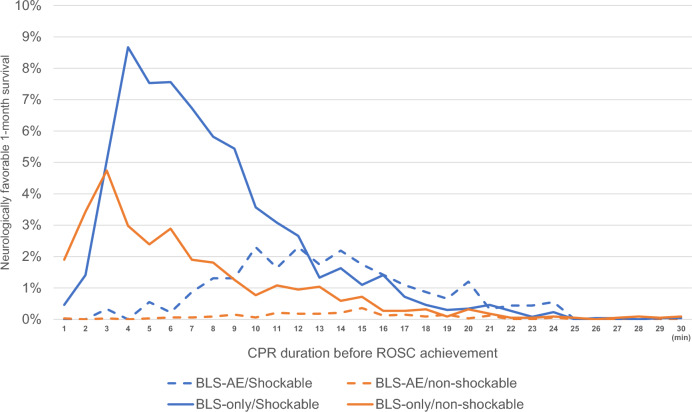
Survival rate for every minute among 4 subgroups. *CPR* cardiopulmonary resuscitation, *ROSC* return of spontaneous circulation, *BLS* basic life support, *AE* administered epinephrine.

In the BLS-only group, patients with shockable and non-shockable rhythms showed the highest survival rate (8.7% and 4.7%, respectively) when the duration between CPR and ROSC was 4 min and 3 min, respectively, with the survival rate rapidly decreasing beyond this point (Fig. 3).

### Factors associated with early ROSC achievement

Based on the ROC curve analysis results (Fig. 2), we defined early ROSC achievement as within 9 min and 6 min for patients with shockable and non-shockable rhythms, respectively. As shown in Table 2, multivariable analysis indicated that BLS-only resuscitation had the most significant impact on early ROSC achievement in patients with shockable [odds ratio (OR; 95% confidence interval (CI)) 9.95 (8.22–12.03) and non-shockable [28.95 (23.08–36.32)] rhythms.

**Table 2:** Factors associated with early ROSC in the multivariate analysis.

Factors	Interval of CPR-to-ROSC	
	≦ 9 min for shockable rhythm	≦ 6 min for non-shockable rhythm
	Odds ratio (95% confidence interval) for early ROSC	
Patient' age		
For every 1yrs increase	0.99 (0.99–1.00)	0.99 (0.98–0.99)
Patient' sex		
Male	0.93 (0.77–1.13)	0.90 (0.76–1.07)
Female	Reference	Reference
BCPR		
Provided	1.50 (1.26–1.78)	1.20 (1.02–1.41)
Not provided	Reference	Reference
Interventions		
BLS-only	9.95 (8.22–12.03)	28.95 (23.08–36.32)
BLS-AE	Reference	Reference
Time factors, for every 1 min increase		
EMS response time	0.97 (0.94–1.01)	0.95 (0.92–0.98)
Interval of collapse-to-CPR	0.99 (0.97–1.01)	0.99 (0.98–1.00)
On-scene time	0.94 (0.92–0.95)	0.95 (0.94–0.97)

*ROSC* return of spontaneous circulation, *CPR* cardiopulmonary resuscitation *BCPR* bystander cardiopulmonary resuscitation, *BLS* basic life support, *AE* epinephrine administration, *EMS* emergency medical service.

Interval of Collapse-to-CPR was defined as the time from cardiac arrest being witnessed by a bystander to the initiation of CPR by EMS.

### Factors associated with neurologically favorable outcomes

Supplemental Table 1 shows the characteristics of patients with OHCA after propensity score matching (BLS-only group: n = 2696 and BLS-AE group: n = 2696). As shown in Table 3, after propensity score matching, the multivariable analysis revealed that the neurologically favorable 1-month survival was associated with BLS-only resuscitation [6.44 (5.34–7.77)], patient age [0.95 (0.94–0.96)], male sex [1.40 (1.17–1.66)], bystander CPR [1.54 (1.32–1.82)], initial shockable rhythms [3.00 (2.56–3.53)], transportation to level-3 hospitals [1.46 (1.25–1.70)], collapse-to-CPR interval [0.97 (0.96–0.99)], CPR-to-ROSC interval [0.95 (0.94–0.97)], and on-scene time [0.96 (0.95–0.98)].

**Table 3:** Multivariate analysis before and after propensity score matching.

Factors	Propensity score matching	
	Before	After
	Odds ratio (95% confidence interval) for survival	
Day of the week		
Weekend (Sat. to Sun.)	1.08 (0.95–1.22)	1.11 (0.94–1.34)
Weekday (Mon. to Fri.)	Reference	Reference
Time of the day		
Nighttime (23:00–6:59)	1.04 (0.89–1.21)	1.11 (0.92–1.34)
Daytime (7:00–22:59)	Reference	Reference
Patient' age		
For every 1 year increase	0.95 (0.95–0.96)	0.95 (0.94–0.96)
Patient' sex		
Male	1.36 (1.18–1.55)	1.40 (1.17–1.66)
Female	Reference	Reference
BCPR		
Provided	1.51 (1.33–1.72)	1.54 (1.32–1.82)
Not provided	Reference	Reference
Initial ECG rhythms		
Shockable	2.99 (2.65–3.38)	3.00 (2.56–3.53)
Non-shockable	Reference	Reference
Interventions		
BLS-only	6.51 (5.56–7.62)	6.44 (5.34–7.77)
BLS-AE	Reference	Reference
Level of hospital		
Level 3	1.47 (1.31–1.65)	1.46 (1.25–1.70)
Level 2 or 1	Reference	Reference
Time factors, for every 1 min increase		
EMS response time	0.96 (0.94–1.00)	0.97 (0.95–1.00)
Collapse-to-CPR	0.98 (0.97–0.99)	0.97 (0.96–0.99)
CPR-to-ROSC	0.95 (0.94–0.96)	0.95 (0.94–0.97)
On-scene time	0.96 (0.95–0.98)	0.96 (0.95–0.98)
Transport time	0.99 (0.98–1.00)	0.99 (0.97–1.00)

*BCPR* bystander cardiopulmonary resuscitation, *ECG* Electrocardiogram *BLS* basic life support, *BLS-AE* basic life support with administered epinepheline, *EMS* emergency medical service, *CPR* cardiopulmonary resuscitation, *ROSC* return of spontaneous circulation.

## Discussion

In this study, our findings indicated that administering BLS for 9 min and 6 min resulted in the best ROSC achievement as outcomes for patients with shockable and non-shockable rhythms, respectively. Moreover, the highest neurologically favorable survival rates were yielded when ROSC was achieved within 4 min and 3 min for patients with shockable and non-shockable rhythms, respectively, in the BLS-only subgroup. Finally, we confirmed that BLS-only resuscitation had the strongest correlation with early ROSC achievement and neurologically favorable outcomes.

The survival rate of patients undergoing resuscitation showed an initial increase followed by a decline after a few minutes^3,14–16^. Another previous study reported that the optimal on-scene time interval for resuscitation was 10 and 8 min in patients with shockable and non-shockable rhythms, respectively^14^. However, these previous studies notably included patients who underwent BLS with or without epinephrine. In our study, the BLS-AE group had a lower survival rate than the BLS-only group for patients with both shockable and non-shockable rhythms. Other studies have demonstrated diminished benefits of epinephrine if administered more than 10 min after the arrival of the BLS providers, and the combination of BLS and epinephrine administration is associated with the best outcomes; however, the studies did not consider the effect of the initial rhythms^17,18^. Based on our findings, we believe that an intervention strategy focusing on BLS should only be implemented for approximately the first 5–6 min after making contact with the patient. If ROSC is not achieved within this timeframe, immediate epinephrine administration is considered an effective intervention strategy.

In this study, the BLS-only intervention, used as a reference to BLS-AE, was associated with early ROSC (≤ 9 min and ≤ 6 min for patients with shockable and non-shockable rhythms, respectively). Notably, in the non-shockable cases, especially those treated with BLS-only intervention, compared to BLS-AE, a higher odds ratio for early ROSC achievement was observed. Guidelines for adult patients with cardiac arrest recommend prompt epinephrine administration to those with non-shockable rhythm^10^. Although it remains unclear at what time in the resuscitation process the decision to transport a patient with ongoing CPR should be made, several studies have recommended minimizing on-scene time and hastening transportation in cases without prehospital ROSC^19,20^. Therefore, to achieve ROSC, we suggest that the EMS should provide high-quality BLS within a shorter timeframe in non-shockable patients than in shockable patients. If this cannot be accomplished, epinephrine administration and transportation should be promptly initiated.

The results of multivariable analysis after propensity score matching revealed that factors associated with favorable 1-month survival were consistent with those in previous studies, including younger age, male gender, provision of bystander BCPR, patients with a shockable initial rhythm, shorter on-scene time, and transportation to a higher-level hospital^21–23^. An additional insight from this study was the significant impact of nighttime cardiac arrests on survival. However, recent meta-analyses have presented contrasting views. The study focused on non-cardiac origin arrests in individuals aged 18 and above, specifically targeting cases without unwitnessed events, cases where ROSC was achieved, and cases without on-scene physician involvement. The deviation from previous research results could be attributed to the differing study populations.

This study has several limitations. First, the findings may not be universally applicable. The median time interval between CPR initiation and epinephrine administration was 11 min, which was longer than the recommended guidelines in Japan^10^ and other regions. This suggests that instances involving the BLS-AE group encompass situations in which patients did not respond to achieve ROSC with BLS only. Indeed, the BLS-AE group had several unfavorable factors compared to the BLS-only group, including older patient age, lower frequency of BCPR provision, longer EMS response times, and a lower rate of shockable initial rhythm. If a similar investigation were conducted in regions where the time to drug administration initiation is shorter, it could potentially yield different results.

Second, the level of hospital to which patients are transported varies depending on the region and EMS protocols. Furthermore, the variations in emergency medical systems and healthcare levels pose an additional potential obstacle to generalizability. Furthermore, our study did not delve into the treatment procedures that patients experienced post-hospital admission. There is a possibility of yielding different results if these aspects were included, deviating from the conclusions drawn in this study.

Third, due to the retrospective nature of the present study, analyzing the quality of chest compressions was not feasible with our available data. Consequently, it remains unclear how the quality of chest compressions may have influenced the outcomes between the BLS-only group and the BLS-AE group. This aspect requires validation through prospective studies.

Lastly, based on the results of the ROC curve analysis, the highest survival rates for patients with shockable and non-shockable rhythms were observed when the time from CPR to ROSC was 4 min and 3 min, respectively. However, the AUC for each was lower than expected (0.6). It might be necessary to narrow down the cases to draw optimal ROC curves that enhance the survival rates for each patient.

## Conclusion

This retrospective study revealed that BLS-only intervention had a significant impact in the initial minutes following CPR initiation. Nevertheless, its effectiveness markedly declined thereafter. The optimal duration for effective BLS-only intervention varied depending on the patient's initial rhythm. Consequently, advanced interventions should be administered within the first few minutes to counteract the diminishing effectiveness of BLS-only intervention.

### Supplementary Information

Supplementary Table 1.

## Data Availability

The datasets used and/or analyzed during the current study are available from the corresponding author on reasonable request.
