# Supplementary material for: Optimal duration and timing of basic-life-support-only intervention for patients with out-of-hospital cardiac arrest
Source: Sci Rep. 2024 Mar 13;14:6071. doi: 10.1038/s41598-024-56487-3 (PMC10937976; doi:10.1038/s41598-024-56487-3)
Supplement: Supplementary file 1 — Supplementary Table 1. [file 41598_2024_56487_MOESM1_ESM.docx]

Supplemental Table 1. Characteristics of OHCAs after propensity score matching

| Factors | | BLS-only | | | | BLS-AE | | | | *p*-value |
| --- | --- | --- | --- | --- | --- | --- | --- | --- | --- | --- |
|  |  | (n | = | 2,696 | ) | (n | = | 2,696 | ) |  |
| Day of the Week | |  |  |  |  |  |  |  |  | 0.745 |
|  | Weekend (Sat. to Sun.) | 816 | ( | 30.3% | ) | 827 | ( | 30.7% | ) |  |
|  | Weekday (Mon. to Fri.) | 1,880 | ( | 69.7% | ) | 1,869 | ( | 69.3% | ) |  |
| Time of the Day | |  |  |  |  |  |  |  |  | 0.890 |
|  | Nighttime (23:00 to 6:59) | 516 | ( | 19.1% | ) | 520 | ( | 19.3% | ) |  |
|  | Daytime (7:00 to 22:59) | 2,180 | ( | 80.9% | ) | 2,176 | ( | 80.7% | ) |  |
| Patient' age | |  |  |  |  |  |  |  |  |  |
|  | median (25–75%) | 76 yrs. (67–84) | | | | 76 yrs. (67–84) | | | | 0.802 |
| Patient' sex | |  |  |  |  |  |  |  |  |  |
|  | Male | 1,831 | ( | 67.9% | ) | 1,828 | ( | 67.8% | ) | 0.930 |
|  | Female | 865 | ( | 32.1% | ) | 868 | ( | 32.2% | ) |  |
| BCPR |  |  |  |  |  |  |  |  |  | 0.868 |
|  | Provided | 1,613 | ( | 59.8% | ) | 1,607 | ( | 59.6% | ) |  |
|  | Not provided | 1,083 | ( | 40.2% | ) | 1,089 | ( | 40.4% | ) |  |
| Initial ECG rhythms | |  |  |  |  |  |  |  |  | 0.502 |
|  | Shockable | 853 | ( | 31.6% | ) | 876 | ( | 32.5% | ) |  |
|  | Non-shockable | 1,843 | ( | 68.4% | ) | 1,820 | ( | 67.5% | ) |  |
| Level of hospital | |  |  |  |  |  |  |  |  | 0.530 |
|  | level 3 | 1,288 | ( | 47.8% | ) | 1,265 | ( | 46.9% | ) |  |
|  | level 2 or 1 | 1,408 | ( | 52.3% | ) | 1,431 | ( | 53.1% | ) |  |
| Time factors, median (25–75%) | |  |  |  |  |  |  |  |  |  |
|  | EMS response time | 8 min ( 7–10) | | | | 9 min ( 7–10) | | | | 0.060 |
|  | Interval of Collapse-to-CPR | 10 min ( 7–14) | | | | 11 min ( 8–14) | | | | 0.002 |
|  | Interval of CPR-to-ROSC | 7 min ( 4–12) | | | | 15 min (12–19) | | | | < 0.001 |
|  | Interval of CPR-to- Epinephrine | - | | | | 10 min ( 7–14) | | | | - |
|  | Interval of Epinephrine -to-ROSC | - | | | | 5 min ( 3– 9) | | | | - |
|  | On-scene time | 12 min (10–16) | | | | 13 min (10–16) | | | | 0.481 |
|  | Transport time | 11 min ( 7–15) | | | | 11 min ( 8–15) | | | | 0.174 |

OHCA, out-of-hospital cardiac arrest; BLS, basic life support; BCPR, bystander cardiopulmonary resuscitation.

ECG, Electrocardiogram, EMS, emergency medical service; CPR, cardiopulmonary resuscitation.

ROSC: return of spontaneous circulation.

The EMS response time was defined as the time from EMS call to arrival at the patient.

Collapse-to-CPR interval was defined as the time from cardiac arrest being witnessed by a bystander to the initiation of CPR by EMS.

On-scene time was defined as the EMS arrival at the patient to the departure of an ambulance.

Transport time was defined as departure from an ambulance to arrival at a hospital.
